# Sexual experience in patients with coronary heart disease: a descriptive phenomenological study

**DOI:** 10.1038/s41598-024-60496-7

**Published:** 2024-04-30

**Authors:** Fengpei Zhang, Yachai Li, Zhili Shi, Aiping Wang

**Affiliations:** 1 Public Business Department, The First Affiliated Hospital of China Medical University, Shenyang, China; 2 Department of Cardiovascular Ward, The First Affiliated Hospital of Xinxiang Medical University, Xinxiang, China; 3 Nursing Department, The First Affiliated Hospital of Xinxiang Medical University, Xinxiang, China

**Keywords:** Coronary heart disease, Sexual experience, Phenomenology, Qualitative research, Cardiovascular diseases, Human behaviour, Sexual dysfunction

## Abstract

Sex is an essential part of life and is a basic demand for human beings. Coronary heart disease can have an impact on patients’ sexual lives; however, not much attention has been paid to it in China, and few studies have been conducted on this topic. Therefore, this study used a qualitative approach to understand the sexual experience of patients after the illness, thus laying the foundation for the development of relevant measures. Semi-structured interviews were conducted with 14 patients. Descriptive phenomenological methods were employed to collect data and explore the sexual experience of patients with coronary heart disease. A total of 4 thematic groups, 9 themes, and 23 subthemes were extracted. The four thematic groups were independent and cross-influenced. In these groups, alterations in the sexual experience, including the change in sexual physiology and psychological state, were affected by the lack of knowledge, age, disease, and other factors. Furthermore, the patient’s perception of sexuality affected the quality of sexual life after the illness. The sexual experience of patients with coronary heart disease and its influencing factors, such as age, disease factors, and lack of related knowledge, were described. The findings are expected to aid in formulating targeted and personalized intervention measures.

## Introduction

According to the “China Cardiovascular Health and Disease Report 2022,” owing to the prevalence of unhealthy lifestyles in China, the presence of cardiovascular disease risk factors, and the acceleration of population aging, the incidence of and mortality associated with cardiovascular disease are increasing. Two out of five deaths are from cardiovascular disease. It is estimated that the number of patients with cardiovascular disease in China is 330 million, of which 11.39 million have coronary heart disease. The mortality rate of coronary heart disease has witnessed an upward trend since 2012^1^. Coronary atherogenic heart disease refers to coronary artery atherosclerosis, lumen stenosis, obstruction, and (or) coronary artery functional changes (spasm) caused by myocardial ischemia, hypoxia, or necrosis, collectively known as coronary arterial heart disease, coronary heart disease, or ischemic heart disease^2^. Coronary heart disease is one of the most common cardiovascular diseases and has become an important illness that poses a serious threat to human life and health.

With the improvement in health awareness, people pay more and more attention to the quality of life after the illness. Sex is a basic demand of human beings and is an essential part of life. The quality of sexual life affects the quality of life of patients.

However, sex is a physical activity, and the emotional and mental excitement resulting from it can cause accelerated respiration and heart rate, increased blood pressure, and enhanced oxygen consumption in the body, which may induce the recurrence of adverse cardiac events^3^. After the illness, the spouse worries about the recurrence of the disease, and the patient is anxious about sexual life, thus restraining or suppressing the needs. This suppression not only affects the marital relationship but also makes the patient and the spouse feel anxious, depressed, and less confident^4^. In addition, the disease itself and the drugs used to treat it, such as antihypertensive drugs, thiazide diuretics, and statins, can affect the patient’s sexual function^5^. Owing to the characteristics of the disease and the sensitivity of sexual topics, patients lack relevant knowledge on sexual life during the recovery period and are also ashamed to seek guidance from medical staff. This inhibition leads to the suppression of sexual needs and even puts an end to sexual life. The quality of sexual life in patients with coronary heart disease is affected by several factors, which are not only physiological but also psychological, social, disease-related, etc. Presently, research conducted in other countries on this topic is mostly quantitative studies that are focused on issues such as patients’ sexual function, sexual satisfaction, and sexual frequency^5–7^. In China, owing to cultural influence, there are few studies on this topic, most of which are focused on the sexual life of male patients and those who have undergone percutaneous coronary intervention (PCI)^3,8,9^. Few researchers have paid attention to the sexual experience of patients after the disease. Therefore, it is necessary to examine the sexual experience of patients with coronary heart disease to deeply understand the current situation and quality of their sexual life and determine the influencing factors. The findings are expected to provide a basis for the development of evaluation tools to enhance the quality of sexual life of patients with coronary heart disease and the formulation of personalized intervention measures.

## Materials and methods

### Descriptive phenomenology

Descriptive phenomenology was proposed by Husserl and further developedby Merleau-Ponty. Husserl advocated “returning to the essence of things.” Using a series of methods such as reduction and suspension, the researcher intuitively faced the facts themselves, described the inner experience of things, and returned to the essence of facts to the greatest extent so as to grasp the facts themselves^10–12^. In this study, descriptive phenomenology was adopted to describe the life experience, i.e., sexual life experience of patients with coronary heart disease. Semi-structured, face-to-face interviews were conducted to comprehensively describe the sexual life of patients with coronary heart disease and understand the factors affecting it.

### Study subjects and samples

Patients with coronary heart disease in the cardiovascular ward of the First Affiliated Hospital of Xinxiang Medical University were selected for the interview. Inclusion criteria: ① Age ≥ 18 years and having a stable sexual partner; ② having a sexual life before the disease; ③ rehospitalized patients with coronary heart disease whose condition was stable; ④ clear mind, no communication disorder, and complete language expression ability; ⑤ normal cognitive function; ⑥ Informed consent and voluntary participation. Exclusion criteria: ① having mental diseases; ② serious acute disease or acute onset of chronic disease. Fifteen patients participated in the semi-structured interview, of which one patient dropped out because of a change in his condition. Two patients were interviewed twice. Hence, a total of 14 patients were finally included in the study. There were 8 women and 6 men, aged 44–84 years, with an average age of 61 years. The shortest duration of the disease was 6 months, and the longest was > 20 years (Table 1).

**Table 1:** Demographic data of this study patients.

No.	Gender	Age	Marital status	Educational level	Disease time (years)
P1	Female	71	Married	Junior	20
P2	Female	66	Married	Senior	9
P3	Female	84	Married	Undergraduate	2
P4	Female	65	Married	Junior	1
P5	Female	51	Married	Senior	1
P6	Male	60	Married	Senior	9
P7	Male	74	Married	Junior	26
P8	Male	51	Married	Junior	0.5
P9	Female	51	Married	Junior	5
P10	Female	68	Married	Senior	4
P11	Male	61	Married	Junior	1
P12	Male	51	Married	Junior college	17
P13	Female	66	Married	Senior	20
P14	Male	44	Married	Junior	0.5

### Sampling method

Purposive sampling was used to select patients who met the inclusion criteria and were likely to provide rich information. Working in the ward provided an opportunity for the researchers to contact several patients with coronary heart disease. After striking a rapport with the patients, the study was described to them and they were invited to participate in it. The snowball strategy was used in this study. After the interview, the patients were requested to introduce the study to other patients they were familiar with.

The sample size was based on repeated themes during the interview. During the progress of the interview, when no more new themes appeared, i.e., when the data saturation was reached, the sample size was finalized.

### Study instruments

#### Sociodemographic characteristics questionnaire

After a literature review and group discussion, information on gender, age, marital status, education level, family income, and disease duration (year) was included in the sociodemographic characteristics questionnaire.

#### Interview outline

According to the purpose of this study, an initial interview outline was formed based on literature reading and group discussion. Two patients who met the inclusion and exclusion criteria were preinterviewed using the initial interview outline. Based on the results of the preinterview, the interview outline was modified to form the final outline (Table 2). A total of 14 patients were interviewed using the final interview outline.

**Table 2:** Interview outline of this study.

No.	Questions
1	Please describe the difference in the relationship before and after the illness
2	Do you have any sexual impulses or sexual demands since the illness? If yes, how often?
3	Do you have a sexual life after the illness? If yes, please talk about your feelings
4	What are the changes in your sexual life after the illness?
5	Could you describe your current sexual life?
6	What factors do you think affect your current sexual life? How do you meet your sexual needs currently?
7	Tell me about your specific views on your current sexual life

## Research team

This study included one female professor with rich experience in qualitative research and two PhD candidates (one female and one male) with experience in qualitative research. The professor in the team was responsible for the project design and quality control, and the two PhD candidates were responsible for data collection and analysis.

### Data collection

In the process of data collection, to ensure participant comfort, the interviews were conducted in a private and undisturbed environment. At the beginning of the interview, the patient was informed that the interview was likely to last for 50–60 min. To ensure data integrity, the researchers used two recorders for audio recording. During the interviewing of each patient, notes were taken on the observed data at all times. Within 24 h after the interview, the notes on each patient were converted to detailed and rich descriptive data. After each interview, the researchers wrote a reflective diary and invited the participants to share the data and analytical results in a timely manner for correction and reflection.

### Data analysis

In this study, the Nvivo software was used to code the data, and Colaizzi’s seven-step method^13^was used to analyze it. The researchers read the source material word for word, adopted the attitude of “surrender” and “suspend” presuppositions and values, extracted the phrases or sentences linked to the research phenomenon, and marked the repeated words and sentences. Two researchers repeatedly reviewed the meaningful words and sentences in the data and summarized them into meaningful units for coding. The codes were classified according to the frequency of relevant words or content, the results were integrated, and the research phenomenon was described in detail. The detailed description was reduced, the theme was refined, and the structural framework was formed according to a certain order and theme.

### Rigor

Reliability, confirmability, credibility, and transferability of the research rigor were tested using Guba and Lincoln standards^14^. In terms of credibility, the researchers entered the cardiovascular ward, participated in the nursing work, interacted with the participants, and established a good relationship with them to collect rich data. The researchers wrote the reflective diary, recorded the impression of the interview process, and verified the contents in the next interview to present the entire interview process candidly and restore the truth of things. In terms of reliability, two researchers transcribed and checked the audio recordings. In the event of a disagreement, a third researcher was consulted. In terms of confirmability, the results of the analysis were fed back to the participants to verify the authenticity and accuracy of the results. In terms of transferability, two qualitative research experts outside the research group were invited to confirm the data.

### Ethical approval

Before commencing the study, the participants were informed about its purpose and significance. Their consent was obtained, and each participant signed an informed consent form. This study was conducted in accordance with the Helsinki Declaration and was approved by the Ethics Committee of The First Affiliated Hospital of Xinxiang Medical University (No: EC-022-005). All participants consent and each participants signed an informed consent.

## Results

**Figure 1:**
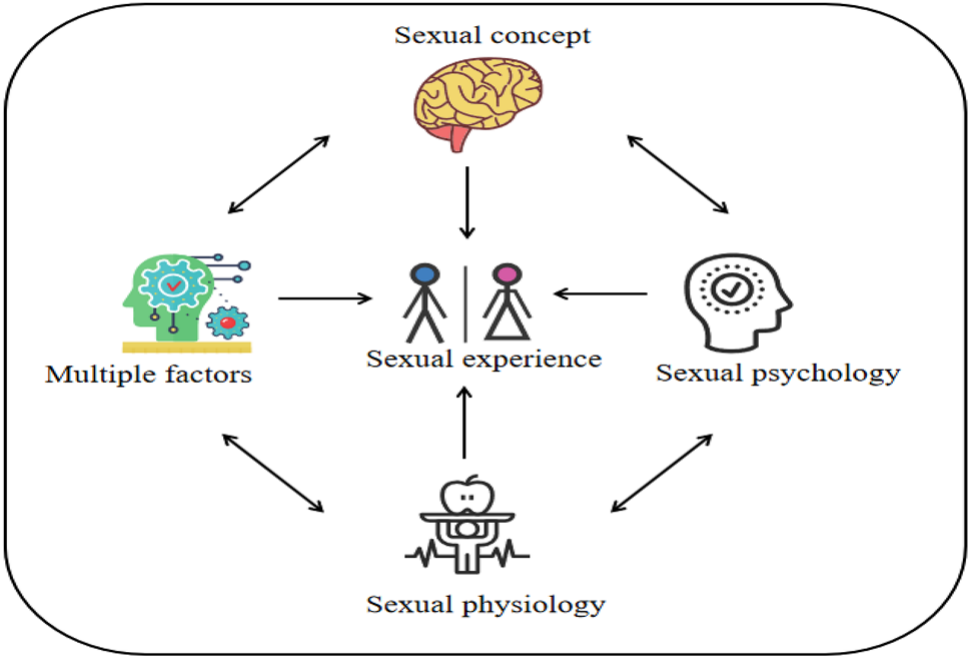
Relationship between themes.

In this study, 4 thematic groups, 9 themes, and 23 subthemes (Table 3) were extracted. The four thematic groups were not only independent of each other but also cross-influenced each other (Fig. 1). In the four thematic groups, alterations in sexual experience, such as the change in sexual physiology and sexual psychology, were represented. Sexual experience was affected by the lack of knowledge, age, disease, and other factors. In addition, participants’ cognition of sexuality affected the quality of sexual life after the illness.

**Table 3:** The theme of this study.

Theme group	Theme	Subtheme
Sexual physiology	Sexual needs	Demand reduction
		Disinterested
		Frequency reduction
	Sexual function	Erectile dysfunction (Male)
		The duration of intercourse is shorter
		Sexual discomfort (Female)
		Unable to do what one hopes to do
Sexual psychology	Fear	Fear of disease recurrence
		Fear of sudden death
	Anxious	Lack of confidence in sexual ability
		Ambivalence
		Care about the spouse’s feelings
Sexual concept	Personal concept	Promote the relationship
		Mutual support
	Social concept	Ashamed to mention
		Life pressure
Influencing factor	Age	Physiological change
		Physical change
	Disease factors	Activities without endurance
		The spouse suffers from illness
		A variety of diseases with patient
	Lack of relevant knowledge	Drug knowledge
		Safe sexual life

### Sexual physiology

**Figure 2:**
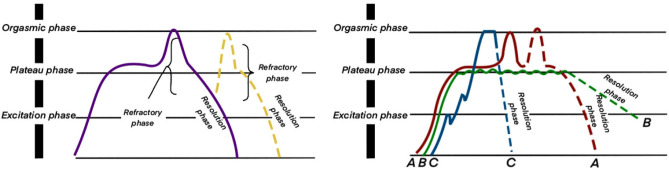
Sexual response cycle^15^ (Left panel: the male sexual response cycle; right panel: the female sexual response cycle).

The sexual physiological response cycle of the normal population comprises the excitation, plateau, orgasm, and resolution phases (Fig. 2). At each stage of the sexual response cycle, the sexual organs undergo corresponding changes. During the interview, alterations in the patient’s sexual response cycle were reported, which reflected changes in sexual organs and response patterns. For instance, during the excitation phase, because of male erectile dysfunction, a stronger stimulation was required to achieve erection. The duration of maintaining an erection became shorter, and premature ejaculation occurred during orgasm. Women needed more stimulation to be sexually aroused or even lost their sexual needs. Some female participants reported a decrease in vaginal fluid, vaginal dryness, and even pain during intercourse.

#### Sexual needs

##### Demand reduction

After the illness, most patients felt that the sexual need was not as obvious as that before the illness and there was also a complete lack of need, so the frequency of sexual activity was reduced.

P(participant)7: Before the illness, I often had thoughts of intercourse with my partner. After the illness, I feel that I have no idea anymore. I think it has an impact on sexual life.

##### Disinterested

One of the patients stated that after the illness, he had no interest in sexual life and no idea of sexual activity.

P12: Not long before getting heart disease, sexual needs were less. Although there were sexual needs after getting sick, I was not interested. I have sexual intercourse occasionally, but I feel that the penis does not become erect and needs stronger stimulation to achieve erection, so I have no interest in sexual intercourse.

##### Frequency reduction

The frequency of sexual activity reflects the quality of sexual life, and after the illness, the frequency was reduced, which affected their sexual experience.

P10: After the illness, the frequency of sexual activity is less. Before the disease, I had sexual intercourse 3–4 times a month. However, after the disease, I have not had sexual intercourse for a long time, and I do not know why. Now, I feel that my sexual needs are significantly less.

#### Sexual dysfunction

Male patients reported erectile dysfunction during sexual intercourse, which became shorter, and the quality of sexual life was not as good as that before the illness. Female patients experienced pain during intercourse, the overall experience was bad, and they even stopped having sex.

##### Erectile dysfunction

Male patients experienced erectile dysfunction in the process of sexual intercourse, and even after stimulation, the duration of the erection was short.

P12: After getting sick, I have an idea about sexual intercourse, but I always feel that the penis does not become erect, and even if it is erect, it does not stay that way for long, and I feel that there is a problem with sexual function.

##### Shortened duration of intercourse

After the illness, the duration of each sexual intercourse became shorter, and sexual life was difficult.

P8: After getting sick, it is not easy to have a sexual life. Ejaculation occurs soon after penetration, and the intercourse time is not as long as that before the illness.

##### Sexual discomfort

Female patients felt vaginal dryness, painful intercourse, and other uncomfortable symptoms, which worsened their sexual experience.

P2: After getting coronary heart disease, sexual life is less than that before the illness. Even if I have a sexual life, I feel uncomfortable as the vagina is dry. I feel pain and a burning sensation and have even stopped having intercourse with my husband.

##### Unable to do what one hopes to do

Sexual dysfunction leads to patients looking forward to sexual life and they feel that their sexual ability is limited, which worsens their sexual experience.

P14: There was no such situation before; we had sexual intercourse and quickly achieved erection. But for nearly half a year, my erectile function has been affected by heart disease (sexual intercourse causes angina symptoms). I think that sometimes I’m not able to achieve an erection because I’m too tired; however, at times, even when I’m not tired, I’m not able to achieve an erection. My erectile function is not as good as it used to be. Sometimes, the penis becomes erect, but it does not last long, and I do not have the mood for intercourse. Sometimes, I feel old, but I am not too old to think about it. Some people are 50 or 60 years old and have a sexual life. I am not able to do it now, and I feel very distressed.

### Sexual psychology

The patients had a sexual life after the illness, but there were some changes in their psychological state and most of the energy was focused on treatment and recovery from the disease. The patients had to wait for the body to fully recover before indulging in sexual activity. But in their sexual life, the couple continued to have concerns, such as “fear, even fear of sex.” They became extremely cautious during sexual life, afraid of inducing cardiac accidents or even sudden death during sexual intercourse. Some patients also felt “anxious” about having a sexual life. They complained that when it came to sexual life, they were “nervous” and felt that their sexual ability was not good and that they were not able to meet the needs of their spouses. At the same time, the spouse’s worry also made the patient feel pressure. Even if the patient had needs, the spouse refused to have a sexual life, so the patient felt “have no other way.”

#### Fear

Most patients are worried about the onset of cardiac accidents and also about sudden cardiac death.

##### Fear of disease recurrence

P9: Just after the PCI, I’m so scared that I do not have a sexual life. I’m afraid of having a heart attack during sexual intercourse with my husband.

##### Fear of sudden death

P2: We had sexual intercourse once in a while. I heard that some heart patients died suddenly during sexual intercourse, so I was scared. Sometimes, I feel uncomfortable having sexual intercourse, so stopped having sexual intercourse.

#### Anxiety

The patients had anxiety about their sexual function, and the tension of the spouse made the patient feel anxious.

##### Lack of confidence in sexual ability

P11: I feel embarrassed to talk about sexual matters with medical staff, but I am incompetent, and I am very upset.

##### Ambivalence

P10: Several times, I have experienced the need for sexual intercourse, but I really have no energy, and I am worried that my husband is feeling unhappy, so I feel very anxious.

##### Care about spouse’s feelings

P8: My wife is very worried about my physical condition. Every time we have a sexual life, she thinks a lot and is always afraid of this and that, which makes me nervous.

### Sexual concept

In Chinese culture, the sexual concept is categorized into two types^16^: individual sexual concept and social concept. In the course of the study, it was found that for patients, personal and social concepts interacted with their understanding of sex. At the individual level, sexual life is more about intimacy and intimate behavior rather than just sex. From the social concept, owing to the influence of social culture, some patients are ashamed to find solutions for their sexual problems and tend to avoid the topic of sexual life. In addition, because of the stress of life, patients lack the energy to focus on sexual problems.

#### Personal concept

##### Strengthen the relationship between the husband and wife

Certain patients believe that a harmonious sexual life plays a positive role in promoting marital relationships, and some of them have a deep understanding of sexual intercourse.

P2: I don’t think sexuality is a shame, and I don’t talk about it every day like young people. I think a harmonious sexual life can enhance the relationship between husband and wife and make the relationship more harmonious.

P3: When I reach a certain age, I think sexuality is not just about sexual intercourse but more about emotional communication, mutual love, mutual care, mutual tolerance, and understanding.

##### Correlative dependence

Sexuality is mainly reflected in the mutual respect and understanding between the husband and wife, i.e., psychological dependence.

P2: I think a harmonious sexual life can enhance the relationship between the husband and wife, make the relationship more harmonious, and respect each other, no matter what you do or think about each other. Couples depend on each other, and this dependence is not only external dependence but also psychological dependence.

P3: We are older, and although the sexual life is less than that before, I think it is more of a close relationship. Sometimes, he will say “I love you,” hold my hand, and show other actions. I think this is more of a psychological dependence, and couples cannot be separated from each other.

#### Social concept

##### Ashamed to mention

Owing to the influence of traditional Chinese culture, the Chinese sexual concept is relatively conservative. For the Chinese, sex is a very sensitive and private topic and is rarely mentioned in front of outsiders. Even when there is a problem, people are ashamed to mention it.

P2: I can’t talk about these things to outsiders. A friend secretly told me to use some lubricant, but I’m embarrassed to use it. He often has sexual needs, and I feel annoyed.

P10: Sexuality is an indispensable part of life, but no one talks about it openly. I don’t know what is good and what is bad. In short, I feel that after the illness, it is not the same as that before. I did not dare to ask, and I was embarrassed to mention it.

##### Life pressure

P13: My life is very stressful, and now I have a disease and have to treat it. Hence, I don’t have much interest in sexual life, and occasionally, I have a one-time sexual life.

### Influence of multiple factors

Sexual life is affected not only by age-related factors but also by the impact of the disease. Moreover, the lack of knowledge about when to resume sexual life after the illness and how to have a safe sexual life affects the quality of patients’ sexual lives.

#### Age

Age and aging cause hormone levels to decline, and these changes cause alterations in sexual organs and their function, thus affecting patients’ needs and sexual experiences.

##### Physiological change

P8: I am older now, and my sexual needs are not so much. I have a sexual life occasionally, and it ends soon.

##### Physical change

P12: I feel so old that I don’t have enough energy and always feel tired.

#### Disease factors

Not only the patient’s illness but also the spouse’s poor physical condition affects the quality of sexual life between the husband and wife.

##### Activities without endurance

P13: Since I got coronary heart disease, I feel palpitation when I walk fast and I feel tired when I sweep the floor at home. So, I have less sexual life and feel physically incapable.

##### The spouse suffers from illness

P12: My wife’s health condition is not good, and she often gets sick. Regarding the sexual life, she does not have much demand and my heart function is not good, so the sexual life is less.

##### Presence of various diseases in the patient

P10: I have not only coronary heart disease. I have undergone breast cancer surgery 7 or 8 years ago and have thyroid problems. Over the years, I have taken a lot of drugs and most of my time has been spent in treatment. So, I do not pay much attention to sexual life.

#### Lack of relevant knowledge

Patients lack relevant knowledge and do not know that certain drugs used for the treatment of coronary heart disease have an impact on sexual function, and some of them do not know when they can resume their sexual life after the disease and whether they can have a sexual life.

##### Drug knowledge

P8: I took a lot of drugs after I got sick, but I didn’t pay attention to their side effects. I was told by chance that one of the drugs I took would affect my sexual function. Then, I recalled the difficulties I had in my sexual life after I got sick and wondered if they were related to the drugs I took.

##### Safe sexual life

P14: Before percutaneous coronary intervention, there was an attack of angina pectoris during the sexual life, and we stopped quickly. After the procedure, when I have sexual needs, I’m worried that sexual intercourse may trigger cardiac events. What do I do?

P1: I do not know when I can resume my sexual life after the discharge. After the illness, I did not have a sexual life for a long time, and then I felt that my physical condition was better and gradually began to have a sexual life.

## Discussion

### Actively treat sexual dysfunction

Sexual responses include arousal, orgasm, refractory, and extinction periods. After coronary heart disease, men experience erectile dysfunction, delayed ejaculation, premature ejaculation, and reduced libido. Women face orgasm disorder, sexual interest or excitement disorder, sexual pain, decreased vaginal lubrication fluid secretion, and other symptoms^17^. The findings from this study revealed that most patients with coronary heart disease had sexual dysfunction and that the symptoms were more obvious in men than in women. Men mainly encountered erectile dysfunction, orgasm dysfunction, decreased libido, and decreased sexual intercourse satisfaction. Women, on the contrary, exhibited reduced vaginal lubrication, decreased libido, orgasm problems, and suspicion and fear associated with sexual activity. Owing to the cause of the disease, patients pay more attention to its treatment. Because of the lack of knowledge and the sensitivity of sexual problems, few patients seek help from medical staff. Therefore, medical staff should inform the patients about the impact of coronary heart disease on sexual function and encourage them to treat the problems actively.

### Pay attention to the change in the sexual psychological status of couples after coronary heart disease

Studies have shown that after myocardial infarction, fear of inducing cardiac events often leads to hesitation in sexual activity, and sexual fear is a common complication of myocardial infarction^18,19^. Psychosexual problems are prevalent among patients and their partners, including anxiety, fear, and depression^20^. The results of this study demonstrated that when patients had sexual life after the disease, the couple tended to have fear and worry about accidents during intercourse. Also, patients suffered from anxiety owing to decreased ability to have sexual intercourse. In addition, they were upset as their spouses stopped having intercourse owing to the fear of experiencing heart-related accidents during the activity. These findings agree with those from related studies^20,21^. In the guidelines, it has been suggested that patients’ spouses should also be included in sexual education and counseling^20^. Moreover, attention should be paid not only to the health education of patients’ sexual physiology but also to their sexual psychology. Furthermore, alterations in the psychological state of the patient’s spouse warrant consideration.

### Sex-related education and counseling for patients with coronary heart disease and their spouses

This study observed that patients lacked sex-related knowledge. Awareness about drugs, the appropriate time to resume sexual life after the disease, precautions for safe sexual life, and the influencing factors were lacking. Owing to the influence of traditional Chinese culture, it is difficult for patients to express their sexual problems and they are ashamed to find ways to solve them. Hence, healthcare providers should consider discussing sexual behavior issues with patients, such as safe sexual life, drugs to treat sexual function, and sexual rehabilitation^22^. Sexual counseling and education should cater to the individual needs and concerns of patients and their spouses. Moreover, medical staff should receive education and training to provide appropriate sexual counseling^23^. Considering the uniqueness of this topic, medical staff should provide personalized sexual health education to patients in various ways^9^ so that they can resume their sexual life after the illness in a timely and appropriate manner.

### Sex- and group-based differences in sexual experiences

While emotional intimacy was the sexual goal for women, men focused more on sexual behavior^24^. This study found that in terms of sexual experience, women paid more attention to sexual intimacy and men prioritized sexual physiology. Furthermore, older people were more concerned about alterations in sexual intimacy, and in many cases, nonsexual physical intimacy was as important as sexual physical intimacy^25^. It is evident from the findings that sexual life is not limited to sexual intercourse but also involves the establishment and maintenance of intimate relationships and the expression of intimate behavior. Therefore, interventions pertaining to patients’ sexual lives should not be limited to sexual physiology but should also encompass intimate relationships and the expression of sexual intimacy. Various methods should be used to intervene in the sexual life of patients. Moreover, sex- and group-based personalized methods and contents are required so that the intervention addresses the needs of specific individuals.

### Theme groups are independent but influence each other

The four theme groups not only affected the patient’s sexual experience independently but also acted on it concertedly. Furthermore, the four themes affected each other, forming an interaction cycle. After the disease, in patients with sexual physiology problems, it affects sexual psychology, which in turn changes and acts on sexual physiology, aggravating the problem of sexual physiology, which is consistent with a foreign study^26^. At the same time, sexual concepts also affect sexual psychology and sexual physiology. In Chinese culture, the sexual concept refers to the general understanding and view of sex and includes sexual physiology, psychology, behavior, morality, and civilization. The Chinese believe that the sexual concept is a psychological concept formed under the social and cultural background and is the moral standard based on which people judge sexual behavior. These concepts influence the Chinese understanding of sexual life^16^, which is also aligned with Chinese characteristics. Chinese traditional culture affects the patient’s sexual concepts, and they think that sex is a very private topic. Hence, when they experience the problem, they avoid talking about it, which aggravates the sexual physiological and psychological issues^9^. These, in turn, affect the sexual experience of the patients. In addition, the interview found that age, disease, lack of sex-related knowledge, and other influencing factors not only directly affected the patient’s sexual experience but also indirectly affected it via their sexual physiology and psychology. Therefore, to improve patients’ sexual experience, intervention from multiple perspectives is warranted.

## Conclusion

Using descriptive phenomenology, this study described the sexual experience and the influencing factors, such as age, disease factor, and lack of relevant knowledge, in patients with coronary heart disease. Based on the findings of this study, appropriate measurement tools can be developed to comprehend the sexual life quality of patients with coronary heart disease. Subsequently, personalized interventions can be formulated according to the situation.

## Data Availability

The data in our article can be shared with everyone.

## Code availability

Code is available upon request from the corresponding author.
